# Screening for undiagnosed pancreatic exocrine insufficiency (PEI) in a cohort of diabetic patients using faecal elastase testing and PEI scoring system

**DOI:** 10.1007/s00592-024-02307-z

**Published:** 2024-05-26

**Authors:** V. Parihar, R. Ballester, P. F. Ridgway, K. C. Conlon, J. Gibney, B. M. Ryan

**Affiliations:** 1 Department of Gastroenterology, Tallaght University Hospital, TallaghtDublin 24, Ireland; 2 Department of Clinical Medicine, Trinity College Dublin, Dublin 2, Ireland; 3 Department of Surgery, Tallaght University Hospital and Trinity College, Dublin, Ireland; 4 Department of Endocrinology, Tallaght University Hospital, TallaghtDublin 24, Ireland; 5 Department of Gastroenterology, Letterkenny University Hospital, Letterkenny, Ireland

**Keywords:** Diabetes, Pancreatic exocrine insufficiency, Faecal elastase (FE1), Pancreatic exocrine insufficiency score (PEI-S)

## Abstract

**Introduction:**

Type 1 and type 2 diabetes mellitus (DM) are often accompanied by mild forms of pancreatic exocrine insufficiency (PEI). The prevalence rates of PEI in diabetic patients are unclear and variable depending on the testing modality and the studies published. The clinical consequences of PEI in diabetics are also not well defined.

**Aim:**

We aimed to determine the prevalence of PEI in a diabetic cohort using the faecal elastase-1 (FE-1) assay as a screening test and to validate a patient-reported symptom-based scoring system, the (PEI-S) for diagnosing PEI within this patient population.

**Methods:**

Two hundred and three diabetic patients attending diabetic and gastroenterology outpatients of a university hospital without previously known PEI were recruited for the study. Demographic parameters, PEI score (PEI-S), and glycated hemoglobin (HBA1c) were documented in standardized data sheets, and a stool sample was obtained. A FE-1 value < 200 μg/g and or a PEIS of > 0.6 was used as the screening cut-off for PEI.

**Results:**

One hundred sixty-six patients returned faecal samples. The prevalence of PEI, as measured by low FE-1, was 12%. Smoking was associated with an increased risk of developing PEI in this diabetic population. No other independent risk factors were identified. The PEI-S system did not differentiate between people with diabetes having a normal and low FE1.

**Conclusion:**

12% of this mixed, real-life cohort of type 1 and 2 DM patients had undiagnosed PEI, as defined by an FE-1 score of less than 200 μg/g. While this may appear low, given the rising prevalence of type 2 DM worldwide, there is likely an unrecognized burden of PEI, which has long-term health consequences for those affected. The PEI-S, a symptom-scoring system for patients with PEI, did not perform well in this patient group.

## Introduction

Diabetes mellitus (DM) is caused by a relative or absolute deficiency of insulin secretion resulting in hyperglycaemia. Besides causing life-threatening complications, it can also result in abnormalities of the exocrine pancreatic gland. The latter include fatty infiltration of the pancreas, reduced size of the pancreas and pancreatic exocrine insufficiency (PEI) or dysfunction. The literature to date has reported widely variable prevalence rates of PEI in diabetics. Some studies have reported a prevalence as high as 50% in Type 1 diabetics and 30–50% in Type 2 diabetics [1], whereas other studies have reported prevalence rates as low as 5.4% [2]. A recent meta-analysis of over 3500 patients reported a PEI prevalence of 39% in type 1 and 28% in type 2 diabetic patients [3]. Most studies to date have used measurement of stool faecal elastase-1 (FE-1) levels as a marker of PEI. Some experts call this diabetic exocrine pancreatopathy a novel and distinct histopathological entity that can be readily distinguished from Chronic Pancreatitis (CP) [4].

Pancreatic exocrine function can be measured differently, none of which is without limitations. Direct hormone-stimulated pancreas function tests are invasive and involve endoscopic aspiration of duodenal juices. Quantifying the coefficient of fat absorption (CFA) or its alternative, mixed 13C-triglyceride (13C-MTG) breath test, is the method of choice for diagnosing PEI. However, neither of these tests is widely available in clinical practice due to the cumbersome and, in some cases, invasive nature of the tests [5, 6]. The faecal elastase-1 (FE-1) assay is an enzyme-linked immunosorbent assay that measures pancreatic elastase-1, providing the pancreatic function’s assessment even in the presence of simultaneous enzyme supplementation. FE-1 is highly stable during passage through the intestine and can be measured in stool samples [7]. Measuring faecal elastase is relatively inexpensive and requires less than 1 g of random stool sample, making it a convenient screening test for PEI [8, 9]. As a result, it has been used in recent studies to assess PEI in Diabetes. Values less than 200 mcg/g suggest pancreatic insufficiency, with values less than 100 mcg/g indicating severe insufficiency [10]. The specificity of FE-1 in demonstrating PEI is 90% in cases with severe insufficiency, and the sensitivity is 100%, whereas in cases with mild to moderate pancreatic insufficiency, the sensitivity decreases to 65%. In an analysis of 345 cases of PEI and 312 controls from 6 studies, the FE-1 assay identified patients having PEI with a pooled sensitivity value of 0.96 (95% CI, 0.79–0.99) and specificity value of 0.88 (95% CI, 0.59–0.97), compared to quantitative faecal fat estimation [11]. However, it is essential to understand that the pancreas secretes FE-1 and is only a surrogate marker of PEI [12] and to be mindful when teasing out non-pancreatic malabsorption.

The PEI symptoms (PEI-S) score has been devised to help diagnose PEI, usually in conjunction with other diagnostic tests and methods. It is designed to complement and improve current best practices rather than replace standard clinical and laboratory-based methods of diagnosing PEI. It is unclear if the PEI-S system will help identify PEI in a diabetic population. Still, if it does, this could lead to more efficient, cost-effective utilisation of limited resources with improved patient health outcomes achieved per monetary unit spent [13].

The study aimed to screen for the prevalence of PEI in people with diabetes attending the outpatient department of a tertiary university hospital using the FE1 test to determine differences between the T1 and T2 diabetic cohort and to correlate it with symptoms using a symptom-based PEI questionnaire.

## Material and methods

### Ethics

All procedures performed in studies involving human participants were in accordance with the ethical standards of the institutional and/or national research committee and with the 1964 Helsinki Declaration and its later amendments or comparable ethical standards.

All patients provided written informed consent. The final clinical study protocol and the informed consent form were approved by SJH/TUH Research Ethics Committee [REC REF 3: 2018–12 List 47 [2]; REC REF 2:2018–11 List 43 [1]; REC REF 1: 2018–10 Chairman’s Action [11].

### Recruitment & methods

It was a single-centre, multi-department prospective study. Consecutive patients who fulfilled the criteria were invited and, after informed consent, included in the study. Patients eligible to participate were men and women aged 18 years and above attending outpatient clinics at our institution who had been clinically diagnosed with Type 1 or type 2 diabetes, according to current American Diabetes Association guidelines [14]. A diagnosis of T1 diabetes was made if the patient had diabetes-associated antibodies and was insulin-dependent at diagnosis**.** Information on demographics (age, gender, ethnicity), year of diagnosis, diabetic medications, smoking, alcohol intake, body mass index and most recent level of glycated haemoglobin (HbA1c) was obtained. In addition, patients were asked to complete PEI -a patient-reported outcome questionnaire (PEI-S) [15]. The version of the PEI-S evaluated consisted of 13 items across two domains, with the participants recalling symptoms in the past seven days. Two sections were measured: Sect. 1 [7] consisted of abdominal symptoms, and Sect. 2 [6] regarding bowel movements. Each item used a five-point Likert scale with verbal descriptors. The total symptom score (mean) was calculated for all respondents to provide information to help diagnose PEI as per the questionnaire. A higher score indicated a greater likelihood of diagnosing PEI. As per evidence published, patients with a total symptom score (mean) greater than or equal to 0.60 were consistent with a diagnosis of PEI if the individual did not have a diagnosis of another gastrointestinal condition. [15].The PEI-S was administered along with the Bristol Stool Form Scale (BSFS) to get an objective assessment of stool form, although the BSFS does not form part of the PEI-S [16]. Patients were instructed and provided with a leaflet on collecting faeces samples at home. Patients were requested to collect stool specimens from the formed part of the stool in case of watery consistency. Exclusion criteria were as follows: at the time of study using orlistat or acarbose; previous history of pancreatic disease, gastrointestinal surgery, immunodeficiency, cancer or gastrointestinal condition such as coeliac disease and Inflammatory bowel disease.

### Statistical analysis

We used IBM SPSS statistics for Windows, version 25.0. Armonk, NY: IBM Corp. Student t-test and chi-square test were used where appropriate. The statistical value of *p* < 0.05 was considered statistically significant.

## Results

**Fig. 1:**
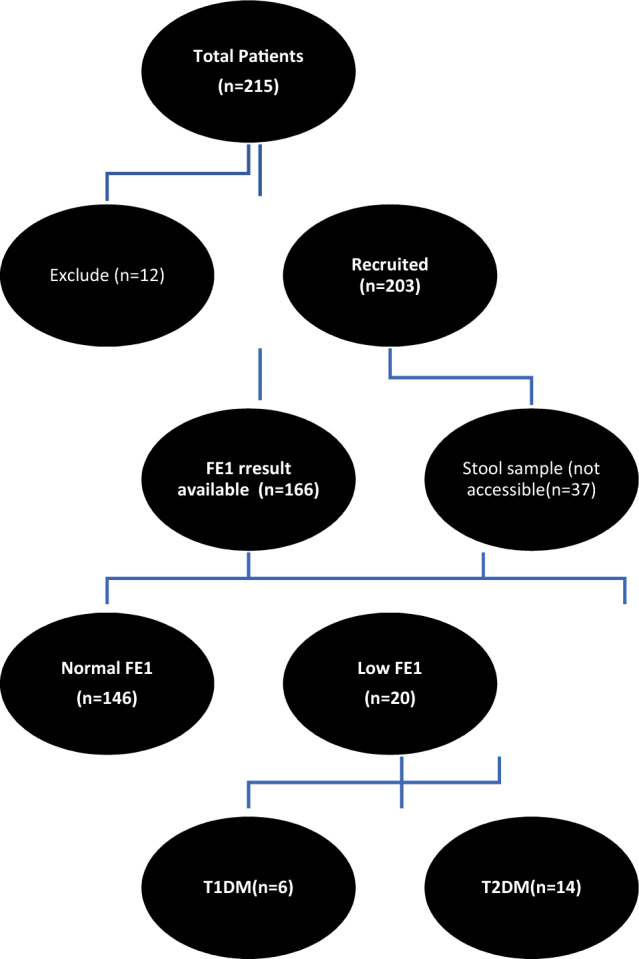
Flow diagram

In total, 203 patients were enrolled in the study. This included 58(28.6%) T1 and 145(71.4%) T2 diabetics. Faecal samples were returned by 166 (81.77%) patients, with 37 patients failing to return the sample pots. The mean age of enrolled patients was 56.54 (18–87) years, with 114(56.2) males. Patients’ characteristics are summarised in Table 1. T1 diabetic patients were younger (42.56 ± 15.21 versus 62.51 ± 11.68 years, *p* < 0.005), with lower BMI (28.04 ± 6.67 versus 32.71 ± 6.58, *p* < 0.005) and longer duration of disease (19.56 ± 12.93 versus 10.40 ± 7.85 years, *p* < 0.005). In addition, they tended to have a higher prevalence of low FE1 (15.8% versus 10.9%, *p* = 0.29) without achieving statistical significance compared to patients with T2DM. There was also a trend of non-Caucasians having more T2DM (*p* = 0.091). The PEI-Q score based on the domains of abdominal pain and bowel movements was marginally higher in T1DM patients without achieving statistical significance regarding the individual constituents and the total score. One hundred and twenty-six (62.1%) of recruits were diagnosed with possible PEI based on the PEI-S results, whereas just 20 (12%) were identified with PEI based on the FE1 test (Fig. 1).

**Table 1:** Baseline demographics of the study population

Variables	All participants	T1	T2	*p*-value
Frequency (percentage)	203	58 (28.6%)	145 (71.4%)	
Age (years)	56.54 ± 15.65	42.56 ± 15.21	62.51 ± 11.68	< 0.005 (Mann–Whitney)
Gender n (%)				
Male	114 (56.2)	32 (28.1)	82 (71.9)	0.490 (Chi-square)
Female	89 (43.8)	26(29.2)	63 (70.8)	
Ethnicity				
Caucasian	176 (86.7%)	55 (94.8%)	121 (83.4%)	*P* = 0.091 Chi-square)
Asian	14 (6.9%)	2 (3.4%)	12 (8.3%)	
Afro-Caribbean	13 (6.4%)	1 (1.7%)	12 (8.3%)	
Source (n %)				
Diabetic clinic	106 (52.2)	42 (39.6)	64 (60.4)	< 0.005 (Chi-square)
GI Clinic	97 (47.8)	16 (16.5)	81 (83.5)	
Follow-up(years)	13.02 ± 10.41	19.56 ± 12.93	10.40 ± 7.85	< 0.005 (Mann–Whitney)
BMI	31.35 ± 6.87	28.04 ± 6.67	32.71 ± 6.58	< 0.005 (Mann–Whitney)
HBA1C	8.37 ± 2.10	8.83 ± 1.96	8.17 ± 2.13	0.007 (Mann–Whitney)
Alcohol consumption(units/week)	7.42 ± 17.41	5.00 ± 8.13	8.29 ± 19.71	0.988 (Mann–Whitney)
Smoker				
Yes	32 (15.8%)	10 (17.2%)	22 (15.2%)	0.858 (chi-square)
No	158 (77.8%)	45 (77.6%)	113 (77.9%)	
Ex	13 (6.4%)	3 (5.2%)	10 (6.9%)

Of the 203 diabetic patients at the time of recruitment, only 2% [4] were taking no medication. The remaining patients were on medication as follows: insulin alone 58 (28.6%), Insulin along with oral agents 37 (18.2%), single 45 (22.2%) and a combination of oral agents 59 (29.1%). 51 (87.9%) of T1 patients were just on insulin monotherapy, whereas T2 diabetics were on variable regimens consisting of single or combination of oral agents and or Insulin. (see Table 2) Of note, the study was carried out before the widespread use of GLP-1 analogues.

**Table 2:** Comparison of diabetics with normal and low FE

	FE1 ≥ 200	FE1 < 200	*p*-value
Number	146	20	
Age	59.23 ± 14.02	56.57 ± 13.05	*p* = 0.281
Sex (M/F)	78/68	12/8	*p* = 0.580
BMI	31.83 ± 6.77	29.44 ± 7.99	*p* = 0.099
Duration	13.29 ± 10.56	13.40 ± 11.86	*p* = 0.872
T1/T2	32/114	6/14	*p* = 0.291
HBA1c	8.31 ± 2.13	8.64 ± 2.01	*p* = 0.363
Ethnicity			
Caucasian	125	16	*p* = 0.424
Asian	10	3	
Afro-Caribbean	11	1	
No of meds			
PEI-S	1.04 ± 0.88	1.18 ± 1.08	*p* = 0.891
PEI-A	1.30 ± 0.96	1.25 ± 1.10	*p* = 0.414
PEI-BM	0.79 ± 0.96	1.12 ± 1.11	*p* = 0.259
Alcohol	6.04 ± 15.32	14.89 ± 28.36	*p* = 0.259
Smoking			
Smokers	32	9	*p* = 0.04
Non-smokers	114	11	

We performed a further sub-analysis of only patients who returned faecal samples (n = 166). Twenty (12%) patients had an FE-1 below the cut-off value. When patients with normal and low FE1 were compared (Table 2), there was no statistically significant difference in the variables checked except in smokers (*p* = 0.04). We divided the patients into groups based on HBA1c, less than or above 8. We analysed the two groups for PEIS (< or ≥ 0.6) and FE1 (< or ≥ 200). There were 158 values available, with 65 (41.1%) with a value of less than 8 and 93(58.9%) equal to or above 8. There was no statistical difference between the two groups, with the p-value being 0.37 in the PEIS group and 0.09 in the FE1 group. The FE1 difference showed that poor glycaemic control increased the trend for PEI but didn’t achieve statistical significance.

Investigating the correlation between PEIS and FE1 level below the cut-off value of 200 µgm/gm, 105 patients with PEIS of > 0.6 ninety -three (88.6%) had normal FE1 while in the case of 12 (11.4%) patients, FE1 was less than 200. This gave PEIS > 0.6 a sensitivity of 0.6 (60%) and a specificity of 0.31 (31%) in diagnosing PEI based on an FE1 level of < 200 µgm/gm in this cohort of diabetic patients. Only 12 individuals with FEV1 less than 200 and PEIS greater than 0.6 were identified among the patients. These patients had an average age of 58.08 ± 12.3, with five of them being males. The average follow-up period for these patients was significantly higher at 15 years. The patients were found to be considerably heavier, with an average BMI of 31.6, and had poorly controlled diabetes with an HbA1c of 8.15. It was observed that three of these patients had T1D. A large percentage (around 68%) of these patients were found to be current or ex-smokers, and 15% of them were consuming excess alcohol.

## Discussion

In this single-centre study, 203 patients were included, with 12 not meeting the inclusion criteria. 29% (58) and 71% (145) had T1 and T-2 diabetes, respectively. T2 patients were older, had increased BMI, and had a shorter duration of the disease than T1 patients, which is typical of the two subtypes of diabetics [17]. This is a regular behaviour cohort, so the results are from a reliable and representative population sample. This is the first study of its kind using FE1 for diagnosing PEI and correlating it with a novel, previously described and validated scoring system in a diabetic population [18].

Pancreatic exocrine insufficiency (PEI) is malabsorption resulting from insufficient digestion of nutrients, especially fats, caused by inadequate secretion of pancreatic enzymes [19]. Clinical symptoms usually occur when pancreatic enzyme activity is severely depleted. These usually consist of steatorrhea, weight loss, excess flatulence, abdominal discomfort and clinical signs of fat-soluble vitamin deficiency [20]. PEI is obviously found in all patients with pancreatogenic diabetes [21] So, our study specifically excluded patients with known CP. Several previous studies using FE1 to assess for PEI in patients with diabetes mellitus reported significantly higher prevalence rates of PEI than in the current study, and this may have been due to the inclusion of patients with Type 3c diabetes [22].

PEI was historically believed to be present in nearly 50% of diabetics tested using direct pancreatic function tests [23] However, when a non-invasive, cheaper FE1 test replaced testing, the PEI rates were shown to be lower [2]. We identified a low FE1 level in 12% of the diabetics in our study. This is comparable to the prevalence of 12.7% reported in another study, with a similar higher prevalence in T1 rather than T2 diabetics. [24]. In yet another study, the prevalence of FE1 below cutoff was only 5.4% [2].

The lower prevalence in our study population is likely due to our strict exclusion criteria. We excluded patients with chronic pancreatitis, for instance, where PEI is known as a common complication. The excellent glycaemic control in patients attending a tertiary hospital as PEI is believed to be a complication of poor glycaemic control [25].This could contribute to our low prevalence as well. Also, some studies have shown that PEI is often mild in some diabetics. [26].Correlation between diabetes duration and the prevalence of PEI is contradictory. A study has suggested that low FE1 correlates with a longer duration of diabetes and higher HBA1c [24]. However, as published in other studies, we found no statistically significant relationship between diabetes duration [27] or HBA1c level and symptoms of PEI or FE1 level below cut off [22, 28]. A study published by Hart et al. in 2000 [29]. Reduced FE1 levels were found in 56.7% of type 1 patients, 35% of type 2 patients, and 18.1% of the controls. The fact that reduced FE1 levels were identified in such a high percentage of normal controls casts some doubt about the validity of the particular assay used.

Data on the occurrence of the symptoms of PEI in diabetic patients are limited. Gastrointestinal (GI) symptoms are common (27–87%) in patients with type 1 and type 2 diabetics [30, 31]. We looked at the clinical relevance of PEI by comparing PEI-S between diabetic patients having normal and low FE1. We found no difference between the two sets of patients, either in overall score or in individual subsets that made up the score. The apparent lack of clinical correlation to the low FE1 was surprising since studies involving other conditions associated with PEI seem to indicate a more unambiguous clinical correlation resulting in impaired quality of life due to steatorrhea, weight loss, abdominal discomfort and other PEI-related symptoms [19]. However, another study did show a lack of clinical correlation to the low FE1 [24]. Several factors could explain the poor performance of the PEI-S score in this diabetic population. The scoring system looks at digestive symptoms; diabetic patients could have digestive symptoms for several reasons –diabetes and associated autonomic neuropathy from the medications, many of which have well-documented GI symptoms or other co-existing conditions. One of them is small intestinal bacterial overgrowth (SIBO), a condition known to be more common among diabetics.

The only association for PEI identified in our cohort was cigarette smoking. This is similar to another study [32]. This suggests that tobacco exposure is an independent risk for pancreatic exocrine insufficiency in patients without an existing diagnosis of pancreatic disease. Tobacco exposure appears to have greater detrimental effects on pancreatic function than alcohol in this population.

The study’s strengths are the large number of patients recruited though predominantly T2 diabetics, normal behaving diabetic cohort, use of a non-invasive diagnostic method and robust exclusion criteria**.** This study’s limitation relates to using FE1 as a diagnostic method due to its low sensitivity in mild to moderate PEI cases. However, in our hospitals and most other institutions, FE1 is the standard test available to check for PEI. The fact that no laboratory-based tests to check for nutritional ill effects of PEI were performed at the time of recruitment would constitute a constraining factor. The study’s statistical power was also limited by the unexpectedly low prevalence of low FE-1. A sample size calculation based on previous studies suggested a sample size of around 132 FE1 (incorporated in ethics application) would be sufficient. A control cohort was envisaged in the research application, but the study was terminated on account of the COVID-19 outbreak before recruitment could start.

## Conclusion

Based on FE1 testing, we identified PEI in 12% of the diabetic patients recruited to this study, with the prevalence being slightly higher in T1 (16%) compared to T2 (11%) diabetics. Smoking is a detrimental factor that may contribute to the development of PEI in diabetics. The PEI-S system did not perform well as a reliable, objective scoring system for diagnosing PEI in this diabetic population. Given the growing number of patients with T2 diabetes in the developed world, a prevalence of 11% of unrecognised PEI amongst these patients correlates with millions of patients with T2 diabetes worldwide who may have undiagnosed PEI. There are over 35 million T2 diabetics in the US and a similar number in Europe, meaning that there could be over 3.5 million diabetics in the US and Europe with undiagnosed PEI. PEI has other health consequences for patients, such as vitamin and micronutrient deficiencies, which may add to the burden of ill health experienced by patients with T2 diabetes. Routine screening for PEI, using FE1 testing, should be considered for all diabetic patients. Furthermore, the finding of a low FE1 should prompt additional investigations on these patients, including diagnostic imaging, as some patients attending diabetic clinics may have unrecognised type 3c diabetes.
